# Comparison between Substance P and Calcitonin Gene-Related Peptide and Their Receptors in Colorectal Adenocarcinoma

**DOI:** 10.3390/jcm13185616

**Published:** 2024-09-22

**Authors:** Robert-Emmanuel Șerban, Mihail Virgil Boldeanu, Dan Nicolae Florescu, Mihaela Ionescu, Mircea-Sebastian Șerbănescu, Lidia Boldeanu, Mirela-Marinela Florescu, Mioara-Desdemona Stepan, Vasile-Cosmin Obleagă, Cristian Constantin, Dragoş-Marian Popescu, Costin Teodor Streba, Cristin Constantin Vere

**Affiliations:** 1 Department of Gastroenterology, University of Medicine and Pharmacy of Craiova, 200349 Craiova, Romania; drrobert.serban03@gmail.com (R.-E.Ș.); cristin.vere@umfcv.ro (C.C.V.); 2 Research Center of Gastroenterology and Hepatology, University of Medicine and Pharmacy of Craiova, 200638 Craiova, Romania; 3 Department of Immunology, University of Medicine and Pharmacy of Craiova, 200349 Craiova, Romania; 4 Department of Medical Informatics and Biostatistics, University of Medicine and Pharmacy of Craiova, 200349 Craiova, Romania; 5 Department of Microbiology, Faculty of Medicine, University of Medicine and Pharmacy of Craiova, 200349 Craiova, Romania; 6 Department of Pathology, University of Medicine and Pharmacy of Craiova, 200349 Craiova, Romania; 7 Department of Infant Care-Pediatrics-Neonatology, University of Medicine and Pharmacy of Craiova, 200349 Craiova, Romania; 8 Department of Surgery, University of Medicine and Pharmacy of Craiova, 200349 Craiova, Romania; 9 Department of Radiology and Medical Imaging, University of Medicine and Pharmacy of Craiova, 200349 Craiova, Romania; 10 Department of Extreme Conditions Medicine, University of Medicine and Pharmacy of Craiova, 200349 Craiova, Romania; 11 Department of Scientific Research Methodology, University of Medicine and Pharmacy of Craiova, 200349 Craiova, Romania; costin.streba@umfcv.ro; 12 Department of Pulmonology, University of Medicine and Pharmacy of Craiova, 200349 Craiova, Romania

**Keywords:** colorectal cancer, neuropeptides, substance P, calcitonin gene-related peptide, neurokinin 1 receptor, calcitonin receptor-like receptor

## Abstract

**Background:** Colorectal cancer is a major health problem that still causes many deaths worldwide. Neuropeptides, such as substance P and calcitonin gene-related peptide, play the neurotransmitter and neurohormone roles that increase tumor invasiveness and metastasis potential. This study aimed to see whether these neuropeptides and their receptors—neurokinin 1 receptor and calcitonin receptor-like receptor—correlate with the diagnosis stage, tumor differentiation grade, and different patient characteristics in colorectal cancer and also to compare them. **Methods:** We performed serum analyses of substance P and CGRP levels in patients with colorectal cancer and also the immunohistochemical analysis of their receptors in colorectal tumors and then correlated them with the disease stage and with different tumor characteristics. **Results:** We demonstrated that both substance P and calcitonin gene-related peptide had increased levels in colorectal cancer and that their levels correlated with the stage of the disease and with the tumor differentiation grade. We also demonstrated the correlation of NK-1R and CRLR higher immunohistochemical scores with advanced and poorly differentiated tumors. **Conclusions:** This study demonstrates that the neuropeptides SP and CGRP and their receptors NK-1R and CRLR could play a role in the pathogenesis of colorectal cancer, and they could be used as diagnostic and prognostic markers and could represent potential therapeutic targets.

## 1. Introduction

Colorectal cancer is one of the leading causes of cancer-related illness and death. At the European Union level in 2022, it represented the second cause of cancer in women and the third in men, also representing the second cause of cancer death [1]. In the United States, it represents the third cause of cancer death [2]. Several risk factors are associated with colorectal cancer: obesity, sedentary lifestyle, excessive consumption of red meat, smoking, heavy alcohol consumption, diabetes, and family history of colorectal cancer [3,4,5,6,7].

The nervous system plays an important role in the pathogenesis of cancer, influencing several processes at the level of the tumor microenvironment, like the regulation of the immune response, the local inflammation, modulation of tumor growth, stimulation of angiogenesis, and dissemination of tumor cells through perineural invasion [8,9,10]. The importance of the nervous system in colorectal cancer is higher, considering the numerous nerve cells at the intestinal level, represented in particular by the enteric nervous system [11,12]. An important component of the role of the nervous system in cancer is represented by neurotransmitters—chemical substances that help transmit information between neurons. Neurotransmitters are involved in the progression of different types of cancer and include not only classic neurotransmitters such as catecholamines, serotonin, acetylcholine, glutamate, and histamine but also neuropeptides like substance P, cholecystokinin, neuropeptide γ, calcitonin gene-related peptide, and bradykinin [13,14,15].

Neuropeptides are substances formed by various numbers of amino acids, which are secreted mostly by neurons, and, besides the role of neurotransmitters, they also play the role of neurohormones and neuromodulators [16]. In cancer, their roles are fulfilled through different mechanisms like the stimulation of tumor progression by tumor cell proliferation, stimulation of angiogenesis, cell migration, and development of metastases [14,17]. Two important neuropeptides in inflammation modulation are substance P and calcitonin gene-related peptide; both have a role in the colorectal cancer pathogenesis, directly or through the persistence of risk factors present since childhood, such as obesity and metabolic syndrome [18].

Substance P is an 11-amino-acid neuropeptide, secreted mainly by neurons but also by immune cells and endothelial and epithelial cells [19,20,21]. Substance P is encoded by the TAC1 gene on chromosome 7 and is part of the tachykinin family together with neurokinin A and neurokinin B, neuropeptide γ, and neuropeptide K [21,22,23,24]. Tachykinins perform their biological role by binding with three receptors: neurokinin 1 (NK-1), neurokinin 2 (NK-2), and neurokinin 3 (NK-3). They are part of the G protein-coupled receptor (GPCR) family and are specific membrane receptors [25,26]. Substance P activates the NK-1 receptor and has roles in various inflammatory processes by stimulating the proliferation of lymphocytes, macrophages, and monocytes; the production of immunoglobulins; and the secretion of cytokines [27,28,29,30]. It also has a strong vasodilator and antiapoptotic role and is involved in various pathologies such as asthma, intestinal inflammatory diseases, and arthritis [31,32,33,34]. SP and NK-1 are involved in tumor progression processes, stimulating the proliferation of tumor cells and their migration, angiogenesis, and metastasis, and have high expression in various types of cancer, such as melanoma, brain tumors and laryngeal, breast, prostate, and also colorectal cancer [35,36,37,38,39].

CGRP is a neuropeptide formed by 37 amino acids, secreted especially by neurons of sensory fibers in the central and peripheral nervous system, and is part of the same calcitonin family with amylin and adrenomedullin [40,41]. It has two isoforms (α and β), with the same biological roles. They come from two distinct genes on chromosome 11: CALC I produces αCGRP, and CALC II produces βCGRP [42,43]. Calcitonin receptor-like receptor (CRLR) belongs to the family of G protein-coupled receptors (GPCRs) and together with receptor-activity-modifying protein (RAMP) creates heterodimeric receptors for CGRP (RAMP1) and adrenomedullin (RAMP2 and 3) [44,45]. CGRP also has an important vasodilatory role; a role in suppressing immunity; and a role in regulating the response of macrophages, T cells, and dendritic cells [46,47,48,49]. Among its most important pathophysiological roles is the modulation of pain in migraines and arthritis, and it also has a protective role in cardiovascular pathologies, especially an anti-hypertensive role [50,51,52,53,54,55,56]. CGRP has high levels in cancers, especially in medullary thyroid carcinoma but also in lung and prostate carcinoma [57]. At the tumor level, CGRP is associated with the proliferation and increased invasiveness of tumor cells and metastasis [58,59].

Our study aimed to correlate the serum levels of the neuropeptides substance P and calcitonin gene-related peptide and the immunohistochemical analysis of their receptors NK1R and CRLR with different patients’ characteristics and with the different tumor characteristics in patients with colorectal cancer and to compare their levels to see whether they could be used as biomarkers in diagnostics and prognosis and also the possibility of using them as future therapeutic targets in this pathology.

## 2. Materials and Methods

In this consecutive case series study, 95 patients with colorectal adenocarcinoma were diagnosed at the Craiova County Emergency Clinic Hospital and the Craiova Gastroenterology and Hepatology Research Center between January and September 2022. This study was approved by the Ethics Committee of the University of Medicine and Pharmacy of Craiova, No. 4/21.01.2022. Only 82 patients from the initial 95 met the study inclusion criteria: patients newly diagnosed with colorectal cancer; no personal history of colorectal cancer or other types of cancer; patients who have not undergone or are not currently undergoing chemotherapeutic, immunosuppressive, corticosteroid, or biological therapy; and patients who signed informed consent for inclusion in this study. Also, 30 patients without important health problems were included in this study forming the control group, with a gender and age distribution ratio similar to that of the patients with colorectal cancer.

The patients with suspicion of colorectal cancer underwent a clinical exam, and clinical data and a complete set of blood tests were collected. After that, a colonoscopy with biopsy was performed and also a CT and/or an MRI to assess the diagnostic stage of the disease.

The level of SP and CGRP was quantitatively determined by an enzyme-linked immunosorbent assay (ELISA) test from the patient’s serum. After the blood was collected, it was centrifuged at 3000× *g,* and then the patient’s serum was utilized with reagent kits from RayBiotech (Peachtree Corners, GA 30092, USA) and Elabscience (Houston, TX, 77079, USA), according to the usage protocol.

For the immunohistochemical analysis, the paraffin blocks were sectioned, and serial sections of 3–4 µm stretched on polysine slides were obtained, which were deparaffinized in xylene (30 min, 58 °C), hydrated with ethyl alcohol of decreasing concentrations (100%, 90%, 80%, 70%, 5 min each), and immersed in distilled water (10 min). Next, the antigen retrieval was carried out by the heat-induced epitope retrieval (HIER) method, which consisted of boiling the sections in a microwave in citrate buffer pH6 (20 min), blocking endogenous peroxidase with hydrogen peroxide 10% (10 min), and non-specific blocking with 2% bovine serum albumin (BSA, 60 min). The sections were incubated overnight at temperatures of 4 °C with the primary antibodies, represented by mouse monoclonal antibody anti-human calcitonin receptor-like receptor (CRLR, clone 998820, R&D Systems - Minneapolis, MN 55413, USA) and rabbit polyclonal neurokinin 1 anti-antibody receptor (NK-1R, Novus Biologicals - Toronto, ON, Canada), in dilutions of 1/75. The reactions were visualized with the EnVision™ FLEX+ System (code K8002, Dako—Santa Clara, CA 95051, USA), which included the secondary antibody, with which the incubation was carried out (30 min), and the reaction detection system, which used 3,3′-diaminobenzidine (DAB), which exposed the brown signals. After visualization, the reactions were interrupted by immersion in distilled water, and then the sections were counterstained with hematoxylin (2 min), washed in running water, and hydrated with ethyl alcohol solutions of increasing concentrations (70%, 80%, 90%, and 100%; 5 min each), cleared in xylene (30 min) and permanently mounted with Canada balsam. To validate the reactions, external positive controls represented by the lung (CRLR, endothelium, mononuclear cells) and the brain (NK-1R, axons, and dendrites) were used.

For the semi-quantitative quantification of the reactions, a classic immunohistochemical score (IS) was used, obtained by multiplying two scores corresponding to the percentage of marked cells and the intensity of the signal [57]. The positivity threshold value of the reactions was 5% marked cells. Thus, for the number of marked cells, the score was 1 (5–25%), 2 (26–50%), 3 (51–75%), and 4 (>75%), and for intensity, the score was 1 (weak), 2 (moderate), and 3 (increased). Final ISs were considered low for values of 1–4 and high for values of 6–12. In this study, the average value of IS per parameter category was included in the range of 2–8. For each case, the reactions were evaluated by two pathologists on 10 × 400 microscopic fields for which average values of the reactions were obtained; in case of inconsistencies, the evaluation was repeated until a consensus was established.

The Motic Panthera DL microscope was used for image analysis and storage.

Statistical analysis was performed with EasyMedStat software (version 3.24), GraphPad Prism 10.3 (GraphPad Software, Boston, MA, USA), and Statistical Package for Social Sciences (SPSS), version 26 (IBM Corp., Armonk, NY, USA). The Shapiro–Wilk test (sample sizes < 50) was used to assess the normality of continuous data. Normally distributed data were assessed through the ANOVA (for multiple groups, with Tukey’s multiple comparisons test being used for post hoc analysis), while Mann–Whitney and Kruskal–Wallis were used when the normality criterion was not fulfilled. Discrete variables were assessed using the chi-square test. A *p*-value < 0.05 was considered significant.

## 3. Results

This study included 82 patients with colorectal adenocarcinoma, aged between 33 and 90 years, with an average age of 71.1 ± 10.34 years; 55 were men and 27 were women. The control group consisted of 30 patients, aged between 44 and 88 years old, with an average age of 70.67 ± 12.14 years; 20 were men and 10 were women. The groups were balanced in terms of age (U = 998.00, z = −1.526, *p* = 0.127) and gender (χ^2^(1) = 3.318, *p* = 0.069).

As we can see in Table 1, the most frequent primary tumor localization was the sigmoid colon, with 28 cases, followed by the ascending colon with 16 cases, the transverse colon with 14 cases, the rectum with 12 cases, and the cecum and the descending colon with 6 cases each.

### 3.1. Patients’ Tumor Characteristics in Colorectal Cancer

After the complete evaluation of the patients, they were sent to surgery or oncological treatment, by case. They were classified according to the UICC TNM classification, with the following results: the most frequent stages were stage II and III with 27 and 26 patients, followed by stage IV with 15 patients, and stage I with 14 patients. Regarding tumor extension, the majority were patients with T3 tumors—49 cases, followed by patients with T4—19 cases, and then T1 and T2 with 7 cases each; 47 of the patients did not have lymph node metastasis (N0), and 35 patients had lymph node metastasis; 10 patients had more than three lymph nodes invaded (N2), and 25 had three or fewer lymph node metastases (N1); 15 patients had distant metastases (M1). Regarding the pathological tumor differentiation grade, 46 patients had a moderate-differentiation tumor (G2), 23 patients had a poorly differentiated tumor (G3), and only 13 patients had a well-differentiated tumor (G1) (Table 2).

### 3.2. Substance P and CGRP in Colorectal Adenocarcinoma

The statistical analysis of the two neuropeptides showed that their level differences were statistically significant for the TNM stage and the pathological differentiation degree (G). For substance P, the level difference for distant tumor metastasis (M) was also statistically significant, but for the tumor extension (T) and lymph node metastasis (N) the results were not statistically significant. For CGRP, the level differences were statistically significant for tumor extension (T), at the limit of statistical significance for distant metastasis (M), and without significance for lymph node metastasis (N) (Table 3).

Both neuropeptides had increased levels compared to the control group from the early stages. For substance P, the smallest difference between consecutive stages was between stage I and stage II (mean diff −0.65), and the biggest difference was between stage III and IV (mean diff −3.39). Substance P was statistically significantly different between different TNM stages; F(3,78) = 5.439, *p* = 0.002. Substance P levels increased from stage I to stage IV, in that order. The Tukey post hoc analysis revealed that the increase from stage I to stage IV (6.007, 95% CI (1.46 to 10.55)) was statistically significant (*p* = 0.005), as well as the increase from stage II to stage IV (5.36, 95% CI (1.42 to 9.30), *p* = 0.003), but no other group differences were statistically significant. For CGRP, the differences were similar between consecutive stages: I and II (mean diff −1.28), II and III (mean diff −0.42), III and IV (mean diff −0.59). CGRP was statistically significantly different between different TNM stages; F(3,78) = 4.184, *p* = 0.008. The CGRP levels also increased from stage I to stage IV, in that order. The Tukey post hoc analysis revealed that the increase from stage I to stage III (1.71, 95% CI (0.10 to 3.32)) was statistically significant (*p* = 0.032), as well as the increase from stage I to stage IV (2.31, 95% CI (0.51 to 4.11), *p* = 0.006), but no other group differences were statistically significant.

Regarding tumor size and extension, substance P had the biggest difference between T2 and T3 tumors (mean diff −1.64), and the smallest difference between T3 and T4 (mean diff −0.21), but the differences between all four groups were not statistically significant; F(3,78) = 1.178, *p* = 0.324. For CGRP, the biggest difference was also between T2 and T3 tumors (mean diff −1.68) and the smallest difference between T1 and T2 tumors (mean diff—0.23). The differences between all four groups were statistically significant; F(3,78) = 3.371, *p* = 0.023. The Tukey post hoc analysis revealed that the increase from T1 to T3 (1.91, 95% CI (0.06 to 3.90)) was statistically significant (*p* = 0.048), but no other group differences were statistically significant.

Regarding lymph node invasion, for substance P the difference was small between N0 and N1 (mean diff −1.28) with a bigger difference between N1 and N2 (mean diff −1.88), but the differences between the three groups were not statistically significant; F(2,79) = 0.719, *p* = 0.491. For CGRP, there were increasing levels for the N1 stage compared to N0 (mean diff −0.33), but a decreased level between N1 and N2 (mean diff −0.33), and the differences between the three groups were not statistically significant; F(2,79) = 0.259, *p* = 0.773.

Both substance P (mean diff −4.74, *p* = 0.016) and CGRP (mean diff −1.13, *p* = 0.094) had higher levels for M1 tumors than those for M0.

Both neuropeptides had almost equal levels between well (G1)- and moderately (G2) differentiated tumors (SP mean diff −0.03) (CGRP mean diff −0.03), with significantly higher levels for moderately to poorly (G3) differentiated tumors (SP mean diff −4.10) (CGRP mean diff −1.60). Substance P was statistically significantly different between different G stages; F(2,798) = 6.263, *p* = 0.003. Substance P levels increased from G1 to G3, in that order. The Tukey post hoc analysis revealed that the increase from G1 to G3 (4.14, 95% CI (0.22 to 8.06)) was statistically significant (*p* = 0.036), as well as the increase from G2 to stage G3 (4.10, 95% CI (1.22 to 6.99), *p* = 0.003), but no other group differences were statistically significant. CGRP was also statistically significantly different between different G stages; F(2,798) = 7.078, *p* = 0.001. The CGRP levels increased from G1 to G3, in that order. The Tukey post hoc analysis revealed that the increase from G1 to G3 (1.91, 95% CI (0.40 to 3.42)) was statistically significant (*p* = 0.009), as well as the increase from G2 to stage G3 (1.60, 95% CI (0.48 to 2.71), *p* = 0.003), but no other group differences were statistically significant.

Both substance P and CGRP had higher levels in older patients, with statistical significance. Regarding gender, substance P had higher levels for male patients than for female patients, but CGRP had almost the same levels between genders, which means that it does not correlate with gender, unlike substance P.

**Figure 1:**
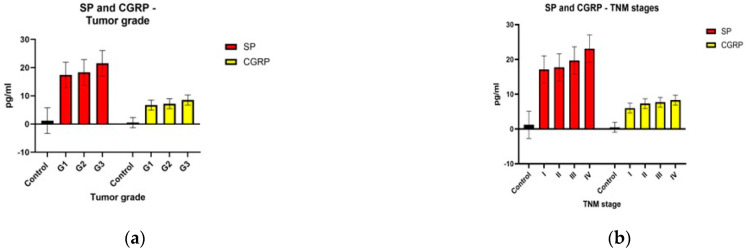

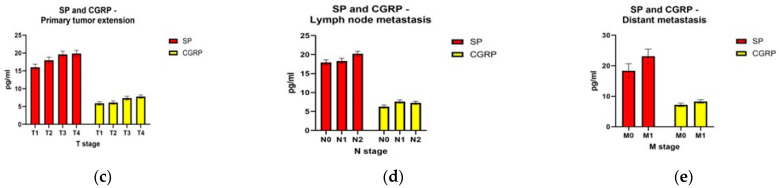
Neuropeptide levels according to pathological tumor differentiation grade—G (**a**); TNM stage (**b**); size and extent of the primary tumor—T (**c**); lymph node metastasis—N (**d**); distant metastasis—M (**e**).

Figure 1 shows graphic representations of the comparisons between the substance P and calcitonin gene-related peptide levels described above.

**Figure 2:**
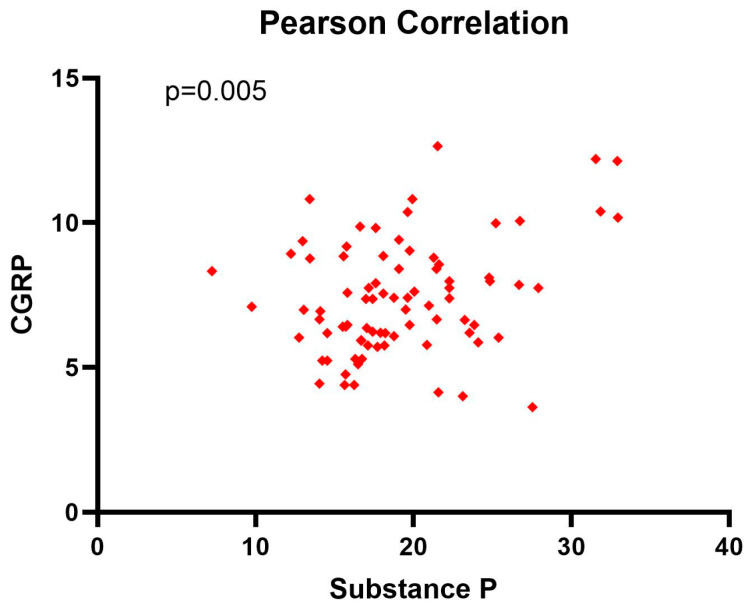
Pearson correlation coefficient between substance P and CGRP levels at diagnosis.

The Pearson (*r* = 0.307, *p* = 0.005) correlation coefficient showed a positive weak correlation between substance P and CGRP, with important statistical significance (*p* < 0.05) (Figure 2).

### 3.3. Comparison between Substance P and Calcitonin Gene-Related Peptide Survival Time in Colorectal Adenocarcinoma

**Figure 3:**
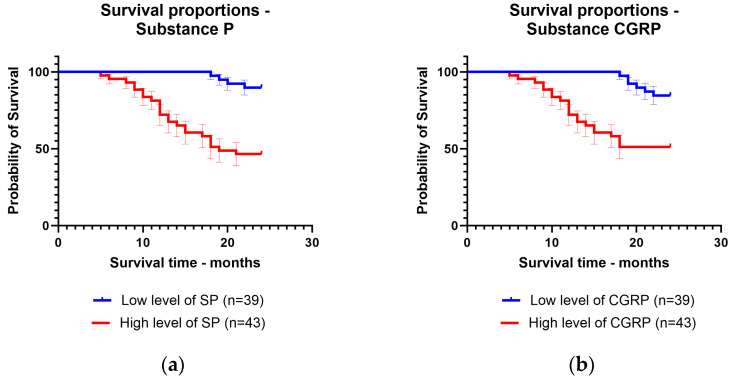
Kaplan–Meier curve for survival time regarding low levels and high levels of: (**a**) substance P; (**b**) calcitonin gene-related peptide.

The patients were followed up for 24 months after the diagnosis. They were divided according to mean levels between patients with low levels of substance P and CGRP and patients with high levels of substance P and CGRP. The mean survival time was 22.82 months for patients with low levels of substance P (92.3% of patients survived) and 17.1 months for patients with high levels of substance P (65.1% of patients survived). For CGRP, the mean survival time was 22.53 months for patients with low levels of CGRP (89.7% of patients survived) and 17.58 months for patients with high levels of CGRP (67.4% of patients survived) (Figure 3).

### 3.4. Immunohistochemical Analyses of Neurokinin 1 Receptor and Calcitonin Receptor-like Receptor

The immunohistochemical analysis of the NK1R and CRLR receptors at tumor levels showed the intense presence of reactions for tumor cells and also for inflammatory cells at the tumor level (lymphocytes, plasma cells, eosinophils, monocytes). The average number of marked cells for NK1R was 59, with an average immunostaining score of 4.7, while for CRLR the average number of marked cells was 55, with an average immunostaining score of 4.5 (Table 4).

The NK1R immunostaining score was higher with advancement in the TNM stages. The immunostaining score was similar for T1 and T2 tumors but higher for T3 and even higher for T4 tumors. Also, the immunostaining score was higher in tumors with lymph node metastasis (N1–2) and with distant metastasis (M1) than in tumors without lymph node or distant metastasis. For pathological tumor differentiation grading, the immunostaining score was higher for poorly differentiated tumors (G3) than for well- and moderately differentiated tumors (G1 and G2) (Table 4).

The CRLR immunostaining score was also higher with the advancement in the TNM stage and for the more invasive (T3, T4) tumors. It was also higher for tumors with the presence of distant (M1) and lymph node (N1–2) metastasis, without a big score difference for tumors with invasion in more lymph nodes (N1 = N2). As in the case of substance P, the immunostaining score was higher for the poorly differentiated (G3) tumors than for the well-differentiated (G1) and moderately differentiated (G2) tumors (Table 4).

**Figure 4:**
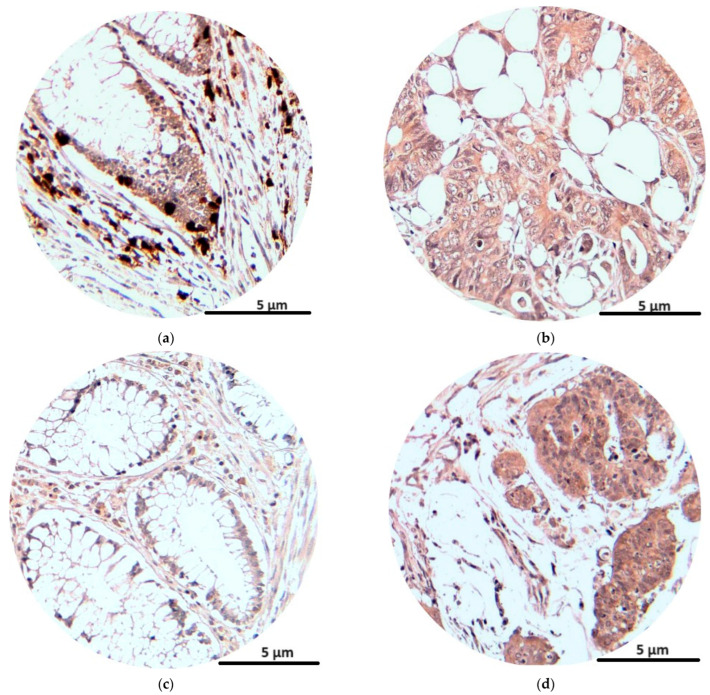
Immunohistochemical staining (magnification × 200): NK1R expression in peritumoral and tumoral tissue (**a**,**b**); CRLR expression in peritumoral and tumoral tissue (**c**,**d**).

In Figure 4, we see the immunostaining reactions of NK1R and CRLR in peritumoral and tumor cells.

## 4. Discussion

The nervous system plays an important role in tumor development. It regulates tumor progression not only through neurotransmitters in a paracrine mode but also by chemical links between the neuron synapses at the nervous system and tumor level like in the case of gliomas [15,60,61]. Also, it may influence tumor progression by modulating the inflammatory response; accelerating the inflammation process; and promoting neurons secreting hormones and peptides that activate cells involved in the immune response such as cytokines, macrophages, and lymphocytes; and also by increasing the angiogenesis process by activating and proliferating endothelial cells. These processes help the migration and metastasis of tumor cells. The nervous system is involved in increasing resistance to treatment, which is performed by inhibiting apoptotic cells, as happens in cervical, breast, and gastric cancer by activating β2-ARs where anti-EGFR treatment and chemotherapy resistance occur [61,62,63,64].

Neuropeptides are a type of neurotransmitter that also can act as neurohormones and paracrine regulators [16]. At the tumor level, they modulate the growth and dissemination of tumor cells, through different mechanisms, such as binding to TRKA and TRKB receptors in pancreatic and breast cancer [65,66]. They also promote angiogenesis by stimulating VEGF expression, like in the case of neuropeptide γ in breast cancer [67]. Substance P and calcitonin gene-related peptides are among the neuropeptides involved in many human physiological and pathological processes, with a proven role in different types of cancers.

Calcitonin gene-related peptide is a neuropeptide from the calcitonin family, formed by 37 amino acids, with two isoforms αCGRP and βCGRP, with a similar biological role [42,43]. CGRP exerts its biological roles by binding to the heterodimer receptor, which is composed of calcitonin receptor-like receptor (CRLR) and receptor activity modifying the protein (RAMP1) [44]. It has a role in the pain process, mostly in migraines [68]. It is a powerful vasodilator that stimulates adenylate cyclase and the production of cAMP and has an effect on regulating blood pressure [50,51]. It also plays a role in dopamine-related nervous system disorders, such as Parkinson’s disease [69,70], and treatment with CGRP monoclonal antibodies alleviates functional abdominal pain, associated with various pathologies, including during the COVID-19 pandemic [71,72].

CGRP and CRLR have not been studied as therapeutic targets until now, either in colorectal cancer or in other types of cancer. There are therapies directed against CGRP and its receptor with proven effects in headache and migraine such as gepants (rimegepant, ubrogepant, atogepant) and monoclonal antibodies (erunumab, fremanezumab, galcanezumab, eptinezumab) [73]. CGRP has increased levels in different types of neoplasm, like medullary thyroid carcinoma, small cell lung cancer, or prostate cancer. Binding with the CLR/RAMP1 receptor increases the invasiveness and migration capacity of tumor cells, promotes epithelial–mesenchymal transition and metastases, and could represent possible therapeutic targets to be included in clinical trials [74,75].

Substance P is a neuropeptide formed by 11 amino acids, from the tachykinin family, which binds with the neurokinin-1 receptor [76]. They have a role in pain expression, including in migraine and also in emesis [77,78].

Substance P is involved in tumor progression in various types of cancers, in which, together with the NK-1 receptors from tumor cells, it increases proliferation, angiogenesis, metastasize, and inhibition of apoptosis through neurocrine, autocrine, or paracrine mechanisms [36,79,80,81]. Several studies show that they are involved in the pathogenesis of nervous system cancers; pancreatic, breast, and lung cancer; melanomas; and also in colorectal cancer [36,82,83,84].

There are effective treatments directed against substance P and the NK-1 receptor. The inactivation of the NK-1 receptor and the blocking of the binding between substance P and NK-1 can be realized by NK-1 receptor antagonists (aprepitant, carsopitant, and orvepitant), which have an anxiolytic and antidepressant effect [85]. Aprepitant and fosaprepitant, by blocking the substance P and NK-1 receptor, have anti-emetic effects in patients after surgery and chemotherapy [80,86,87].

Previous studies showed that blocking NK-1R in colorectal cancer (with aprepitant) induces apoptosis in the tumor cells by inactivating certain signaling pathways and can also increase chemotherapy sensitivity [88]. The advantages of aprepitant, such as wide availability and low price, make this NK-1R antagonist a possible future treatment to be introduced in clinical trials in colorectal cancer.

In this study, we demonstrated that substance P with NK--1R and calcitonin gene-related peptide with CRLR correlate with colorectal cancer. Both neuropeptides had higher serum levels even from the early disease stages than those in the control group patients. They also had higher levels in older patients, which may mean that both neuropeptides may have a more important pathogenic role for this type of patient. Substance P had higher levels in men than in women, meaning that it may have a more important pathogenic role in men. This can also be useful in setting higher cut-off levels in the serological diagnosis of neuropeptides, according to age, and in the case of substance P, according to gender.

Higher levels of neuropeptides correlate with advanced TNM stages, distant metastases, poorly differentiated tumors, and a lower 24-month survival rate. This means that they could have an important diagnostic and prognostic role. For the diagnosis, neuropeptide levels may help us to select and prioritize patients who would need a colonoscopy and imaging for disease staging (CT scan, MRI, etc.) [89,90,91,92] and may also help us to know which patients would have a poorer prognosis and lower survival rate, for the best possible management of these patients. Considering that neuropeptides have increased levels in patients with advanced TNM stages, with more extensive tumors, low-differentiated tumors, and distant metastases, it could mean that together with their receptors, which also have higher staining scores in these patients, they could play a role in and tumor development and invasiveness and also in distant organ metastases. Unlike CGRP, substance P levels also correlate with the number of lymph node metastases in patients with colorectal cancer, which means that it may play a role in lymph node invasion. Considering this, therapies targeted against neuropeptides and their receptors, by blocking their effects, could stop tumor development and distant organ metastases, and in the case of substance P could limit lymphatic metastases. Taking into account the slightly higher immunostaining score of NK-1R in comparison with that of CRLR in colorectal adenocarcinomas, we can say that both substance P and NK-1R correlate more than CGRP and CRLR with the tumoral and clinical-pathological characteristics of patients with colorectal cancer but without significant differences. Both neuropeptides, therefore, can represent diagnostic and prognostic biomarkers in patients with colorectal cancer and together with their receptors could represent important therapeutic alternatives to current oncological therapy in colorectal cancer.

To find the most effective biomarkers for the diagnosis and prognosis of colorectal cancer, comparisons between these molecules are necessary to see which can be the most useful. There are different studies with comparisons between tumor markers, pro-inflammatory cytokines, and other biomarkers in colorectal cancer [93,94,95,96]. It should be noted that a mutual potentiation of each other in tumor development and the extension of the disease cannot be excluded. Bearing in mind the similar levels at the different stages of the disease demonstrated in our study, subsequent studies that are centered on this objective must be performed.

The limitations of this study were the relatively short follow-up period of the patients (24 months), the rather small number of patients, and the fact that they came from a single medical center.

To our knowledge, this is the first article in which the levels of both substance P and calcitonin gene-related peptide are compared and correlated with colorectal cancer.

## 5. Conclusions

The nervous system, along with chronic inflammation, plays an important role in the pathogenesis of cancers, including colorectal adenocarcinoma. Substance P and CGRP are two neuropeptides with high levels in patients with colorectal adenocarcinoma serum and are associated with the diagnosis and prognosis of patients with this pathology. Their increased levels are found in the more advanced stages of the disease and also in more aggressive, undifferentiated tumors. The immunohistochemical analysis at the tumor level showed the important presence of the neuropeptide receptors, NK-1R and CRLR, with higher immunostaining scores in more advanced and poorly differentiated tumors. Substance P and calcitonin gene-related peptide could represent useful diagnostic and prognostic biomarkers in colorectal cancer and, together with their receptors, neurokinin 1 receptor, and calcitonin receptor-like receptor, could represent potential therapeutic targets, but the limitations of this study must be taken into account—the small number of patients, the lack of heterogeneity of the patient group, and the relatively short follow-up period. Extensive multicenter clinical studies with a more heterogeneous population should be performed given the worldwide importance of colorectal cancer.

## Figures and Tables

**Table 1:** Case distribution according to patient characteristics, clinical data, and primary tumor localization in patients with colorectal cancer.

Patients’ Clinical Data		No. of Cases	Total
Mean Age—Colorectal patients	≥71	46 (56.1%)	82 (100%)
	<71	36 (43.9%)	
Mean Age—Control Group	≥71	16 (36.7%)	30 (100%)
	<71	19 (63.3%)	
Gender—Colorectal patients	M	55 (67.1%)	82 (100%)
	F	27 (32.9%)	
Gender—Control group	M	20 (66.7%)	30 (100%)
	F	10 (34.3%)	
Primary tumor site	Rectum	12 (14.6%)	82 (100%)
	Sigmoid	28 (34.1%)	
	Descending	6 (7.3%)	
	Transverse	14 (17.1%)	
	Ascending	16 (19.5%)	
	Cecum	6 (7.3%)	

**Table 2:** Case distribution according to disease stages and tumor characteristics in patients with colorectal cancer.

Colorectal Cancer Characteristics		No. of Cases	Total
TNM stage	I	14 (17.1%)	82 (100%)
	II	27 (32.9%)	
	III	26 (31.7%)	
	IV	15 (18.3%)	
T stage	T1	7 (8.5%)	82 (100%)
	T2	7 (8.5%)	
	T3	49 (59.8%)	
	T4	19 (23.2%)	
N stage	N0	47 (57.3%)	82 (100%)
	N1	25 (30.5%)	
	N2	10 (12.2%)	
M stage	M0	67 (81.7%)	82 (100%)
	M1	15 (18.3%)	
Tumor grade	G1	13 (15.9%)	82 (100%)
	G2	46 (56.1%)	
	G3	23 (28.0%)	

**Table 3:** Substance P and calcitonin gene-related peptide levels depending on patient characteristics and tumor characteristics in patients with colorectal cancer.

Clinical Data and Tumor Characteristics	Substance P (ng/mL) Mean ± SD	*p*-Value	CGRP (ng/mL) Mean ± SD	*p*-Value
Control Group	1.18 ± 0.26		0.45 ± 0.31	
TNM stage		0.002 **		0.008 **
I	17.08 ± 3.58		6.00 ± 1.28	
II	17.73 ± 4.33		7.29 ± 1.72	
III	19.69 ± 4.18		7.72 ± 1.59	
IV	23.09 ± 6.55		8.32 ± 2.73	
T stage		0.324 **		0.023 **
T1	16.20 ± 1.29		5.88 ± 0.87	
T2	17.96 ± 4.92		6.12 ± 1.67	
T3	19.60 ± 5.63		7.36 ± 1.87	
T4	19.82 ± 3.93		7.80 ± 1.98	
N stage		0.491 **		0.773 **
N0	18.30 ± 3.03		7.29 ± 2.04	
N1	18.90 ± 5.65		7.63 ± 1.98	
N2	20.19 ± 4.36		7.30 ± 1.51	
M stage		0.016 *		0.094 *
M0	18.35 ± 4.21		7.19 ± 1.69	
M1	23.09 ± 6.55		8.32 ± 2.73	
Tumor Grade		0.003 **		0.001 **
G1	18.03 ± 3.67		6.68 ± 1.32	
G2	18.07 ± 3.99		6.89 ± 1.92	
G3	22.18 ± 6.35		8.59 ± 1.84	
Age		0.035 *		0.002 *
≥71 years old	19.68 ± 5.78		7.97 ± 2.02	
<71 years old	18.63 ± 3.86		6.65 ± 1.60	
Gender		0.589 *		0.941 *
Male	14.72 ± 9.51		5.55 ± 3.52	
Female	13.72 ± 8.29		5.50 ± 3.56	

* Independent *t*-test. ** One-way ANOVA and Tukey’s multiple comparison test.

**Table 4:** Neurokinin 1 receptor and calcitonin receptor-like receptor mean immunohistochemical score depending on tumor characteristics in patients with colorectal cancer.

Parameter		NK1R IS Score	*p*-Value	CRLR IS Score	*p*-Value
Tumor extension (T)	T1	3.2	0.02 **	2.2	0.01 **
	T2	3.0		3.7	
	T3	5.7		5.1	
	T4	6.5		7.3	
Lymph node metastasis (N)	N0	3.2	0.04 **	3.1	0.01 **
	N1	5.6		6.6	
	N2	6.4		6.8	
Distant metastasis (M)	M0	3.7	0.01 *	3.9	0.01 *
	M1	6.7		7.0	
TNM stage	I	2.9	0.01 **	3.1	<0.01 **
	II	3.7		4.5	
	III	5.2		6.1	
	IV	6.5		7.9	
Tumor pathological grade (G)	G1	2.8	0.01 **	2.6	0.02 **
	G2	4.8		4.2	
	G3	7.4		6.9	

* Mann–Whitney U test. ** Kruskal–Wallis test.

## Data Availability

Data are contained within the article.
